# Neuroplasticity of pain processing and motor control in CAI patients: A UK Biobank study with clinical validation

**DOI:** 10.3389/fnmol.2023.1096930

**Published:** 2023-02-14

**Authors:** Yiran Wang, Qianru Li, Xiao'ao Xue, Xiaoyun Xu, Weichu Tao, Sixu Liu, Yunyi Li, He Wang, Yinghui Hua

**Affiliations:** ^1^Department of Sports Medicine, Fudan University, Shanghai, China; ^2^School of Exercise and Health, Shanghai University of Sport, Shanghai, China; ^3^Department of Biomedical Engineering, Shanghai University of Traditional Chinese Medicine, Shanghai, China; ^4^Institute of Science and Technology for Brain-Inspired Intelligence, Fudan University, Shanghai, China; ^5^Human Phenome Institute, Fudan University, Shanghai, China; ^6^Key Laboratory of Computational Neuroscience and Brain-Inspired Intelligence, Ministry of Education, Fudan University, Shanghai, China; ^7^Yiwu Research Institute of Fudan University, Yiwu, China

**Keywords:** ankle injuries, functional magnetic resonance imaging, functional connection, pain, central nervous system

## Abstract

**Background:**

Pain plays an important role in chronic ankle instability (CAI), and prolonged pain may be associated with ankle dysfunction and abnormal neuroplasticity.

**Purpose:**

To investigate the differences in resting-state functional connectivity among the pain-related brain regions and the ankle motor-related brain regions between healthy controls and patients with CAI, and explore the relationship between patients' motor function and pain.

**Study design:**

A cross-database, cross-sectional study.

**Methods:**

This study included a UK Biobank dataset of 28 patients with ankle pain and 109 healthy controls and a validation dataset of 15 patients with CAI and 15 healthy controls. All participants underwent resting-state functional magnetic resonance imaging scanning, and the functional connectivity (FC) among the pain-related brain regions and the ankle motor-related brain regions were calculated and compared between groups. The correlations between the potentially different functional connectivity and the clinical questionnaires were also explored in patients with CAI.

**Results:**

The functional connection between the cingulate motor area and insula significantly differed between groups in both the UK Biobank (*p* = 0.005) and clinical validation dataset (*p* = 0.049), which was also significantly correlated with Tegner scores (*r* = 0.532, *p* = 0.041) in patients with CAI.

**Conclusion:**

A reduced functional connection between the cingulate motor area and the insula was present in patients with CAI, which was also directly correlated with reduction in the level of patient physical activity.

## 1. Introduction

Lateral ankle sprain is one of the most common sports injuries (McCriskin et al., 2015; Jiang et al., 2018). Although approximately 80% of acute injuries have shown recovery with conservative treatments, the remaining 20% have demonstrated chronic ankle instability (CAI) and have suffered from a range of symptoms (e.g., persistent pain, impaired proprioception, and repetitive re-injury) (Safran et al., 1999; Alghadir et al., 2020). The long-term chronic course can also lead to a reduced quality of life and the development of ankle osteoarthritis (Thompson et al., 2019). Despite a variety of currently utilized conservative treatments and surgery to treat CAI, the outcomes have been far from satisfactory. It is apparent, therefore, that additional research elucidating the pathological mechanisms is essential to deepen our understanding of CAI which could form the basis for more effective clinical intervention (Doherty et al., 2017).

To emphasize the vital role pain plays in CAI, current evidence suggests it is present in 58% of these patients, yet most studies on CAI have continued to ignore pain in their inclusion criteria or primary outcomes (Gribble et al., 2014; Al Adal et al., 2019, 2020). Ankle dysfunction associated with CAI, such as the sense of ankle instability, has been accompanied with pain (Gribble et al., 2014; Al Adal et al., 2020; Xue et al., 2021a). Moreover, persistent pain has been reported to disturb proprioception thereby jeopardizing safe return to sports (RTS) in patients with CAI (Al Adal et al., 2020). However, although pain and the corresponding ankle dysfunction are among the most common symptoms of CAI, existing studies have not thoroughly explained the underlying mechanisms. Recently, a growing number of studies have suggested that long-term pain and dysfunction of musculoskeletal disorders may be associated with maladaptive neuroplasticity in the central nervous system, and we postulated that this upstream mechanism might exist in CAI (Pelletier et al., 2015).

The concept of neuroplasticity suggests that the central nervous system has the ability to adapt to both the external environment and internal factors, which can change the degree of involvement of certain connections or brain areas through different neural circuits (Sharma et al., 2013). In recent years, functional magnetic resonance imaging (fMRI) has gradually shown its value in neurological studies of neuroplasticity, especially the functional connectivity (FC) evaluated by the between-region correlation of the blood oxygen level-dependent (BOLD) signal that reflects the simultaneous nature of blood flow in different brain tissues (Ogawa et al., 1990, 1993). Of particular interest, in musculoskeletal disorders, such as lower back pain and knee ligament injuries, altered FC has been recognized between patients and healthy controls that might negatively affect the time of returning to sports, especially the FC between the pain-related and motor-related areas that are associated with emotional processes, perception of visual motion and body form (e.g., the amygdala, postcentral gyrus, temporal-parietal junction, and middle temporal visual cortex) (Needle et al., 2017; Gandhi et al., 2020; Conboy et al., 2021; Xue et al., 2021b). However, similar evidence of an abnormality relative to FC in CAI has not been demonstrated.

Therefore, we aimed to analyze the functional connectivity between the ankle motor-related and pain-related areas in patients with CAI, to obtain evidence on whether pain is related to motor function and time to return to exercise, based on the initial exploration in patients with ankle pain of the UK Biobank (currently the most extensive human genetic cohort sample library), and further validation in patients with CAI enrolled from a sports medicine clinic. We hypothesized that there was an abnormal functional connection between the pain-brain region and the ankle-motor brain region in patients with CAI, which might also correlate with the clinical outcomes of the patients.

## 2. Methods

### Participants and data acquisition

The study was designed with two parts of cross-sectional analyses to explore the pain symptoms in patients with CAI. Ethical approval was granted for the UK Biobank data usage, and the present research was prospectively registered online (No. 62721). Validation for using the dataset and all study protocols was approved by the Institutional Review Board Huashan Hospital, Fudan University (No. 2016M-008). Informed consent was obtained, and the rights of all subjects were protected.

Part 1 was an initial analysis based on existing MRI data in the UK Biobank (http://www.ukbiobank.ac.uk/). Participants were included if they met the diagnosis based on the ICD10 disease diagnostic classification codes M25.57 Pain in joint (Ankle and foot), and M79.67 Pain in limb (Ankle and foot). Exclusion criteria included patients with painful symptoms due to other factors, joint instability beyond the ankle, no brain imaging, not right-handed, lack of demographic data, and other excluded diseases. Brain images were acquired after the diagnosis of their disease. The diagnosis code of chronic ankle pain and the excluded codes can be found in supplemental digital content (Supplementary Table 1). The final patient group was matched to healthy controls in a 1:4 ratio using sex, age, BMI, and ethnic background.

Part 2 was a validation study using the participants we enrolled from February 2021 to July 2021. CAI patients were recruited by the sports medicine department at the Huashan Hospital, Fudan University. Adult patients were selected if they met the following recommendation of the International Ankle Consortium: (1) history of at least one significant ankle sprain, which occurred at least 12 months prior to the study, resulting in pain, swelling, and at least one interrupted day of normal physical activity; and (2) a score of < 24 on the Cumberland Ankle Instability Tool (CAIT) questionnaire (Hiller et al., 2006; Gribble et al., 2014). Additionally, all participants had been diagnosed with anterior talofibular ligament injuries through physical examinations (positive anterior drawer test) and imaging assessments (ultrasonic or ankle MRI) by an experienced orthopedist (Prof. Yinghui Hua) (Song et al., 2019). Furthermore, all participants were right-footed, defined by the limb they preferred to kick a ball. Exclusion criteria included: (1) a history of other musculoskeletal problems or surgeries of the lower extremities (except the CAI in the patient group); (2) acute ankle sprains or other injuries in the previous 3 months; (3) the medical history of or current medication usage for major medical illnesses, such as cardiovascular, respiratory, neurological, autoimmune, or mental disorders.

### 2.2. Data acquisition

MRI data acquisition protocols of the UK Biobank are presented online (https://biobank.ctsu.ox.ac.uk/crystal/crystal/docs/brain_mri.pdf). For the validation dataset, the Tegner Activity Rating Scale score and American Orthopedic Foot and Ankle Society Score (AOFAS) were measured prior to MRI scanning. The validation dataset MRI scanning was performed on a 7T scanner (MAGNETOM Terra, Siemens Healthcare, Erlangen, Germany) equipped with a 1Tx/32Rx head coil (Nova Medical, Wilmington, MA, USA). Subjects were reminded to lie still at rest, looking at a cross reflected on a projector without thinking about anything. First, a MPRAGE sequence was used to acquire high resolution 3D T1-weighted images (sagittal): TR = 8.1 ms, TE = 3.7 ms; field of view = 256 × 256 mm; matrix = 256 × 256; inplane resolution = 1 × 1 mm; slice thickness = 1 mm; number of slices = 180. Second, fMRI data of resting-state were acquired with the following parameters: TR = 650 ms; TE = 30 ms; flip angle = 53°; field of view = 200 × 200 mm; acquisition matrix = 68 × 66; SENSE factor = 1.5; reconstructed in-plane resolution = 2.5 × 2.5; slice thickness = 3.5 mm; number of slices = 40; multi-band factor = 4. In total, 500 frames of data were collected, and the total time for the resting-state fMRI sessions was approximately 5.5 min.

### 2.3. Data analysis

For preprocessing of the images, Restplus (version 2019) software of Matlab2014a was used, including slice timing and realign, normalization to Montreal Neurological Institute (MNI) template space (resampled to 2 mm isotropic), and smoothing (8 mm full-width half-max kernel). Participants with movement outlier were confirmed using mean frame-wise displacement (FD) >0.2 mm, which would be excluded from the analysis.

For ROIs analysis, 5 mm spheres with combined left and right ankle motor-related regions related to motor were created based on previously published fMRI data of ankle movements, including 9 regions, such as bilateral primary sensorimotor cortex (SM1), supplementary motor area (SMA-proper), cingulate motor area (CMA), premotor cortex (PMC), secondary sensory cortex (SII), basal ganglia, and cerebellum (vermis, anterior lobes). The thalamus was excluded because the coordinates of bilateral brain regions were not available (Kapreli et al., 2007). Also, the 159 pre-defined pain-related ROIs (4 mm sphere) were evenly placed out in pain-related brain regions that were selected from a meta-analysis automatically generated from 516 articles labeled with the term “pain” in the framework of neurosynth (www.neurosyntsh.org) (Woo et al., 2014; Flodin et al., 2016) (**Figure 2A**). The volume of the ROI spheres was used to represent the total voxel size of this brain region, and the time series of BOLD signal under this ROI was extracted. The FCs calculated by the Fisher-z transformed Pearson correlation coefficients between the ankle motor-related and pain-related ROIs to identify neural differences in brain connectivity between ankle motor control and pain processing (Diekfuss et al., 2019).

The remainder of the statistical analyses were performed by Graphpad Prism Version 9.0. Data normality was tested using the Kolmogorov-Smirnov test. Chi2 tests, Mann Whitney U-tests, and independent two-sample *t*-tests were applied to examine the equivalence of demographic variables and the differences in clinical features between groups. Independent two-sample *t*-tests were used for comparisons between groups in both parts, and the two-tailed threshold of *p* < 0.01 was used to identify the potentially altered connectivity in the initial exploration of part 1, and the one-tailed threshold of *p* < 0.05 was used to validate the altered connectivity in part 2. Two-tailed Pearson correlation was also used to estimate the correlation between the significantly different FCs and the clinical questionnaires (i.e., AOFAS and Tegner scores) in patients with CAI, with the absolute values of correlation coefficients (r) classified as weak (0–0.4), moderate (0.4–0.7), or strong (0.7–1.0).

## 3. Results

### 3.1. Demographic and clinical features

**Figure 1:**
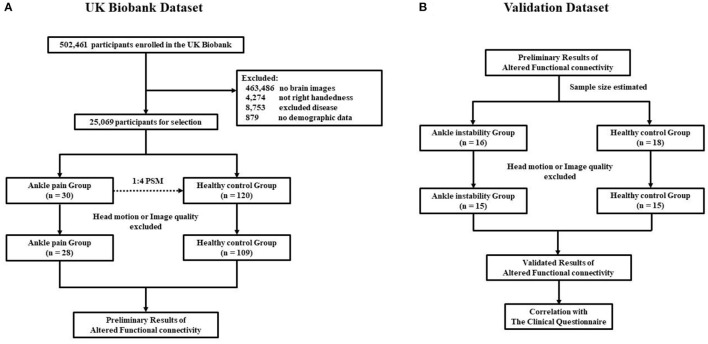
**(A)** Flowchart of the participants selection in part 1; **(B)** Flowchart of the participants selection in part 2.

A total of 502,461 participants were screened from the UK Biobank Resource, of which 463,486 were removed because they had no brain imaging, 4,274 were removed because they were not right-handed, 8,753 were excluded for disease, 879 were removed because they had no demographic data. The remaining 25,069 participants were screened based on the ICD10 disease diagnostic classification. After the exclusion of major illnesses/ traumas, joint instability beyond the ankle, inclusion of ankle instability and selecting fMRI data quality, 28 participants were included in the chronic ankle pain group and 109 in the healthy control group for part 1, the detailed ICD10 codes and diagnosis are presented in Supplementary Tables 1–3. The flowchart of the participant selection in part 1 is presented in Figure 1A. Individual demographic data are shown in Table 1. In Group Chronic Ankle pain, there were 17 females and 11 males, and in Group Healthy Control, there were 63 females and 46 males. No significant differences were observed between the two groups for sex, age, BMI, and ethnic background (*p* > 0.05).

**Table 1:** Demographic variables of ankle pain group and healthy control group presented as mean (SD).

**UK Biobank set**	**Ankle pain (*n =* 28)**	**Healthy control (*n =* 109)**	***P* value**
Sex (female/male)	17/11	63/46	0.949
Age (years)	64.86 ± 7.08	63.93 ± 6.97	0.531
BMI (kg/m^2^)	26.82 ± 4.28	27.13 ± 5.71	0.791
Ethnic background			0.872
A	26 (92.9%)	96 (88.1%)	
B	0 (0.0%)	3 (2.8%)	
C	1 (3.6%)	3 (2.8%)	
D	0 (0.0%)	1 (0.9%)	
E	0 (0.0%)	1 (0.9%)	
F	1 (3.6%)	2 (1.8%)	
G	0 (0.0%)	3 (2.8%)	

In part 2, 16 CAI patients and 18 healthy controls were recruited, while four subjects were excluded due to excessive head motion, leaving 15 in each of the patient groups for further data analysis. The flowchart of participant selection in part 2 is also presented in Figure 1B. Demographic data are shown in Table 2. In Group CAI, there were 2 females and 13 males, and in Group Healthy Control, there were 3 females and 12 males. No significant differences were observed between the two groups for sex, age, BMI, and ethnic background (*p* > 0.05). The CAI patients had significantly worse sports level (Tegner) and ankle function (AOFAS) (*p* < 0.001) when compared with healthy controls.

**Table 2:** Demographic variables of chronic ankle instability group and healthy control group presented as mean (SD).

**Validation set**	**Ankle pain (*n =* 15)**	**Healthy control (*n =* 15)**	***P* value**
Sex (female/male)	2/13	3/12	1.000
Age (years)	26.20 ± 6.12	26.53 ± 2.39	0.846
BMI (kg/m^2^)	24.13 ± 2.26	23.19 ± 2.30	0.265
Ethnic background			1.000
**H**	15 (100%)	15 (100%)	
Tegner scores	3.27 ± 1.71	5.07 ± 1.10	0.002
AOFAS	99.33 ± 2.58	69.87 ± 17.26	<0.001

### 3.2. FC analysis

**Figure 2:**
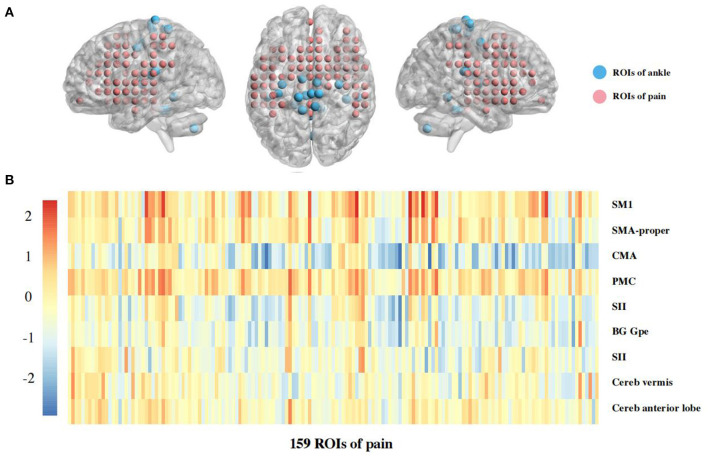
**(A)** The ROIs in global brain as sphere. Each ROI was put in a brain as sphere, for blue color was ROIs of ankle motor regions, and for red color was ROIs of pain regions. **(B)** Heatmaps of functional connectivity between 9 ROIs of ankle activity and 159 ROIs of pain in the global brain. Warmer colors (red and yellow regions) indicate stronger connectivities whereas cooler colors (blue and green regions) indicate weaker connectivity. The 9 ROIs associated with ankle activity are primary sensorimotor cortex (SM1); supplementary motor area (SMA-proper); cingulate motor area (CMA); premotor cortex (PMC); secondary sensory cortex (SII); basal ganglia (BG); external segment of globus pallidus (Gpe); cerebellum (Cereb), in which signsificant differences presented between CMA and Insula (*p* = 0.005), CMA and Putamen (*p* = 0.004), CMA and Rolandic Operculum (*p* = 0.004), SII and Putamen (*p* = 0.009). These ROIs were validated in part 2 of this study.

**Figure 3:**
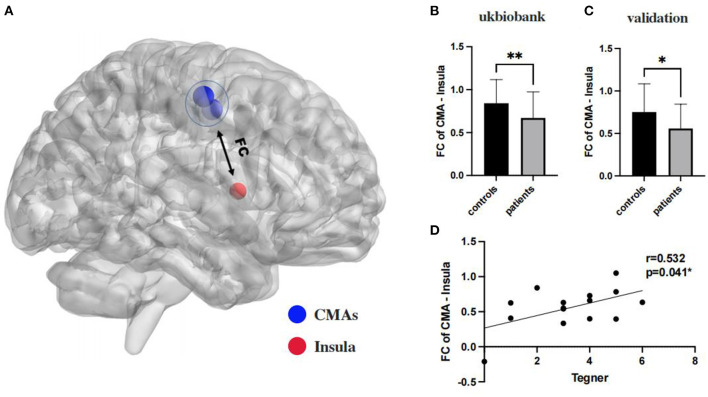
**(A)** The insula and CMAs ROIs in global brain as sphere, in which blue color was ROIs of CMAs, and for red color was ROIs of Insula; **(B, C)**: the functional connectivity between CMA and insula are significant on a corrected cluster level in ukbiobank (*p* = 0.005) and clinical validation (*p* = 0.049) both; **(D)**: functional connectivity of CMA and insulation significantly correlated with Tegner scores (*r* = 0.532, *p* = 0.041). **Significant (*p* < 0.001); *Significant (*p* < 0.05).

A Heatmap of the t value of the between-group comparisons of the FCs between 9 ROIs of ankle activity and 159 ROIs of pain in the global brain is shown in Figure 2B. As compared to group healthy controls, brain FC was significantly lower in group chronic ankle pain in brain ROIs, including CMA and Insula (*p* = 0.005), CMA and Putamen (*p* = 0.004), CMA and Rolandic Operculum (*p* = 0.004), SII and Putamen (*p* = 0.009). These ROIs were used in the validation study in part 2; only the CMA and Insula persisted in patients with CAI (*p* = 0.049) (Table 3, Figure 3). The FC of CMA and Insula had a moderate correlation with Tegner scores (*r* = 0.532, *p* = 0.041), as shown in Figure 3D, while there was no significant correlation with the AOFAS scores (*r* = 0.2738, *p* = 0.3234).

**Table 3:** Comparison of functional connectivity between the group chronic ankle pain and healthy control in two parts of study.

**UK Biobank dataset**	**Ankle pain (*n =* 28)**	**Healthy control (*n =* 109)**	**Direction of difference**	***P* value^#^**
CMA–Insula	0.67 (0.30)	0.84 (0.28)	<	0.005^**^
CMA–Putamen	0.57 (0.31)	0.73 (0.25)	<	0.004^**^
CMA–Rolandic Operculum	0.65 (0.31)	0.83 (0.28)	<	0.004^**^
SII–Putamen	0.50 (0.30)	0.65 (0.27)	<	0.009^**^
**Validation dataset**	**Ankle pain (*****n** =* **15)**	**Healthy control (*****n** =* **15)**	**Direction of difference**	***P*** **value**^%^
CMA–Insula	**0.56 (0.29)**	**0.75 (0.33)**	**<**	0.049^*^
CMA–Putamen	0.59 (0.28)	0.55 (0.41)	>	0.380
CMA–Rolandic Operculum	0.70 (0.25)	0.70 (0.37)	>	0.499
SII–Putamen	0.55 (0.29)	0.45 (0.27)	>	0.186

ns, not significant^**^Significant (*p* < 0.001);

* Significant (*p* < 0.05).

## 4. Discussion

The most important finding of this study was that FC between CMA and insula was reduced in patients with CAI and the degree of connectivity was positively correlated with impaired motor function. To our knowledge, this study is the first fMRI study to evaluate the neuroplasticity of pain processing and motor control in patients with CAI.

The insula, a region involved in pain processing, is usually divided into partial subregions based on anatomy and connectivity (Centanni et al., 2021). Generally, the insula is thought to affect somatosensory, nociceptive, and affective functions (Starr et al., 2009; Segerdahl et al., 2015). Meanwhile, integration of information inputs in the insula provides the prediction of a combination of pain, perceptual errors, or a variety of other current conditions (Geuter et al., 2017; Centanni et al., 2021). The integration of emotional and somatosensory signals is a crucial function of the insula (Gonsalves et al., 2021). Previous studies have confirmed that enhanced nociceptive sensitization in patients with chronic pain may be associated with overlapping pain activation in the insula (Ploghaus et al., 2001; Zhuo, 2016). In addition, reduced pain inhibition is associated with stronger resting-state FC in relation to the insula and other cortical brain regions (Huynh et al., 2022). Furthermore, impaired insula function can also produce or enhance emotions such as anxiety (Paulus and Stein, 2010; Avery et al., 2014; Zhuo, 2016). Therefore, we believe that the insula has a valuable research significance in patients with chronic pain, not only in relation to their pain management but also in terms of predicting their psychological patterns and behavior.

The CMA area is anatomically divided into four regions and is not perfectly aligned in function (Lu et al., 1994; He et al., 1995; Wessel et al., 1995). Numerous studies have demonstrated that the CMA is significantly activated during lower limb movements, even activated before active awareness during voluntary activity (Ball et al., 1999). Increased functional connectivity can be thought of as increased neuronal activity (Lin et al., 2008). In contrast, repetitive somatosensory and motor stimuli, and cognitive stimuli reflect increased resting-state functional connectivity (rsFC) in the brain network, which may be related to the aggregation of neurotransmitter receptors at synaptic terminals (Tung et al., 2013; Wei et al., 2014). Some studies have shown increased rsFC of the CMA with other motor-related cortical brain regions in stroke patients (Liu et al., 2020), which contradicts the results of the present study, but may indicate that the CMA and insula have different patterns from normal activity in processing movements related to pain sensation.

The present study links the insula to the CMA is an attempt to explain its reduced connectivity with decreased motor function. The conclusion that weaker intensity connectivity between the CMA and insula implies that these two brain regions failed to work well together, resulting in a temporal difference of activation in sequence, which forms the theoretical basis for further correlation analysis with pain and ankle dysfunction. We suggest that this reduction in FC may indicate neuroplasticity in CAI patients. It should be noted that if the conflict exists between brain regions during processing information when exercising, this may be the cause of many CAI failures to recover motor function. Due to prolonged chronic pain, patients develop a lower response-ability to pain which in turn adjusts motor behavior. Based on our clinical experience, CAI patients with pain often have a greater fear of rehabilitation. Whether this occurs earlier or later than neuroplastic changes is uncertain, but clearly this psychological change delays their RTS. After participants became aware of their motor intentions, fear of movement-related pain caused hyperactivation in the cingulate motor area. This hyperactivation may result from a conflict between the unrealized desire to avoid the painful experience and the motivation to perform the desired motor task (Osumi et al., 2021). This may in part explain the poorer RTS function and longer RTS times in patients with CAI who experience long-term chronic pain. As some studies have similarly identified a progression of pain sensitization in the follow-up of post-operative CAI patients, the critical role of pain in clinical prognosis is emphasized. Our findings may further explain the process of injury-related outcomes in patients with CAI.

To further investigate the clinical significance of pain in patients with CAI, we assessed the relationship between CMA, insula and clinical scores. Tegner scores were designed to provide a standardized method of assessing the level of motor function through physical activity, and a two-year follow-up of athletes showed that t-scores also provided valid information in predicting RTS function in impaired patients (Klasan et al., 2021). In other words, reduced connectivity of the CMA to the insula directly correlated with the Tegner score. To some extent, it also indicated reduced RTS motor function. However, no significant correlation between rsFC and AOFAS scores was found in this study, which may be principally attributed to the following: (1) AOFAS is used in the clinic mostly to assess patient activity function, and the assessment of pain is less accurate than a VAS; (2) AOFAS is more subjective, and pain perception in different states varies greatly for CAI patients.

Through validation in clinical populations, we found a reduced regional connectivity between the insula and CMA in CAI, but in the UK Biobank data, we also found a significant results between the CMA and putamen, Rolandic operculum, even SII and putamen. This represents an interaction between pain and motor function that does not necessarily exist solely between the CMA and the insula. However, due to the false positives, we were unable to verify all their authenticity, which suggests that future work needs to focus more on the full range of symptoms in CAI patients rather than instability alone.

Current research regarding pain-sensitized brain function has emphasized abnormal connectivity between brain regions, rather than simply abnormal activation of brain regions. These intrinsic connectivity networks offer exciting new avenues for subsequent in-depth research: to perform functional analyses between brain regions in pain and further investigate the presence of neuroplasticity between the “upstream and downstream of pain”. This, in turn, may shift attention to pain-induced central changes such as functional brain connectivity, abnormal activation of associated brain regions, and even large-scale brain network analysis. Concomitantly, we can optimize the clinical questionnaire evaluation system to determine more concordance with rsFC to help predict patient clinical symptoms and psychological status (Gonsalves et al., 2021).

We have also noted that many sports injuries produce pain that is not easily eliminated, so research on the FC between pain sensation and sports injuries can help us understand more about sports injuries. Additionally, due to the limitations of existing treatments, more treatments for pain after sports injuries should be developed. Several studies have shown that TMS can be used to enhance neuroplasticity and reduce pain after injuries (Lu et al., 2015; Shin et al., 2018; Krishnan et al., 2019). It is expected to facilitate more specific interventions in the process of passive assessment of injury-related stimuli to improve future surgical outcomes in patients with cerebral infarction and even other motor injuries.

### 4.1. Limitation

Several limitations should be noted. First, the increased false-positive rate associated with comparisons between multiple ROIs in our exploratory and preliminary study should be acknowledged. However, two sub-studies were performed with inter-validation, which may help to reduce the false-positive rate. Second, although similar to the sample size of previous studies of FC, ours was still smaller. To compensate, we used imaging of two groups, which may have reduced the false positive rate, and the use of a 7T MRI machine can also improve accuracy to a certain extent. Additionally, during data analysis, bilateral foot injuries were combined due to the deficiencies of the UK Biobank itself. The inclusion of more clinical subjects with more detailed clinical evaluations would certainly provide more accurate interindividual variability.

### 4.2. Conclusion

Reduced FC between the CMAs and the insula was present in patients with ankle instability and ankle pain, which was also directly correlated with reduction in the level of physical activity.

## Data availability statement

The original contributions presented in the study are included in the article/Supplementary material, further inquiries can be directed to the corresponding authors.

## Ethics statement

The studies involving human participants were reviewed and approved by Institutional Review Board Huashan Hospital, Fudan University. The patients/participants provided their written informed consent to participate in this study. Written informed consent was obtained from the individual(s) for the publication of any potentially identifiable images or data included in this article.

## Author contributions

YW carried out the study design, data collection, quality rating, statistical analysis, and manuscript writing. XXue carried out the study design, data collection, quality rating, statistical analysis, and manuscript reviewing. QL carried out the study design, data collection, quality rating, and manuscript reviewing. XXu carried out the study design and manuscript reviewing. YH carried out the study design, supervision of the literature search, data collection and quality rating, and manuscript reviewing. All authors have read, approved the final version of the manuscript, and agree with the order of presentation of the authors.

## References

[B1] Al Adal S. Mackey M. Pourkazemi F. Hiller C. E. (2020). The relationship between pain and associated characteristics of chronic ankle instability: a retrospective study. J. Sport Heal. Sci. 9, 96–101. 10.1016/j.jshs.2019.07.009 31921485 PMC6943759

[B2] Al Adal S. Pourkazemi F. Mackey M. Hiller C. E. (2019). The prevalence of pain in people with chronic ankle instability: a systematic review. J. Athl. Train. 54, 662–670. 10.4085/1062-6050-531-17 31184959 PMC6602397

[B3] Alghadir A. H. Iqbal Z. A. Iqbal A. Ahmed H. Ramteke S. U. (2020). Effect of chronic ankle sprain on pain, range of motion, proprioception, and balance among athletes. Int. J. Environ. Res. Public Health 17, 1–11. 10.3390/ijerph17155318 32718066 PMC7432694

[B4] Avery J. A. Drevets W. C. Moseman S. E. Bodurka J. Barcalow J. C. Simmons W. K.. (2014). Major depressive disorder is associated with abnormal interoceptive activity and functional connectivity in the insula. Biol. Psychiatry. 76, 258–266. 10.1016/j.biopsych.2013.11.027 24387823 PMC4048794

[B5] Ball T. Schreiber A. Feige B. Wagner M. Lü C. H. Kristeva-Feige R.. (1999). The role of higher-order motor areas in voluntary movement as revealed by high-resolution EEG and fMRI. Neuroimage. 10, 682–694. 10.1006/nimg.1999.0507 10600414

[B6] Centanni S. W. Janes A. C. Haggerty D. L. Atwood B. Hopf F. W. (2021). Better living through understanding the insula: Why subregions can make all the difference. Neuropharmacology. 198, 108765. 10.1016/j.neuropharm.2021.108765 34461066 PMC13284909

[B7] Conboy V. Edwards C. Ainsworth R. Natusch D. Burcham C. Danisment B.. (2021). Chronic musculoskeletal impairment is associated with alterations in brain regions responsible for the production and perception of movement. J. Physiol. 599, 2255–2272. 10.1113/JP281273 33675033 PMC8132184

[B8] Diekfuss J. A. Grooms D. R. Yuan W. Dudley J. Barber Foss K. D. Thomas S.. (2019). Does brain functional connectivity contribute to musculoskeletal injury? A preliminary prospective analysis of a neural biomarker of ACL injury risk. J. Sci. Med. Sport. 22, 169–174. 10.1016/j.jsams.2018.07.004 30017465 PMC6311430

[B9] Doherty C. Bleakley C. Delahunt E. Holden S. (2017). Treatment and prevention of acute and recurrent ankle sprain: an overview of systematic reviews with meta-analysis. Br. J. Sports Med. 51, 113–125. 10.1136/bjsports-2016-096178 28053200

[B10] Flodin P. Martinsen S. Altawil R. Waldheim E. Lampa J. Kosek E.. (2016). Intrinsic brain connectivity in chronic pain: a resting-state fMRI study in patients with rheumatoid arthritis. Front. Hum. Neurosci. 10, 107. 10.3389/fnhum.2016.00107 27014038 PMC4791375

[B11] Gandhi W. Rosenek N. R. Harrison R. Salomons T. V. (2020). Functional connectivity of the amygdala is linked to individual differences in emotional pain facilitation. Pain. 161, 300–307. 10.1097/j.pain.0000000000001714 31613866

[B12] Geuter S. Boll S. Eippert F. Büchel C. (2017). Functional dissociation of stimulus intensity encoding and predictive coding of pain in the insula. Elife. 6, e24770. 10.7554/eLife.24770.016 28524817 PMC5470871

[B13] Gonsalves M. Beck Q. Fukuda A. Tirrell E. Kokdere F. Kronenberg E.. (2021). Mechanical Affective Touch Therapy (MATT) for anxiety disorders: effects on resting state functional connectivity. Brain Stimul. 14, 1630–1631. 10.1016/j.brs.2021.10.137 35088729 PMC9256848

[B14] Gribble P. A. Delahunt E. Bleakley C. M. Caulfield B. Docherty C. L. Fong D. T. P.. (2014). Selection criteria for patients with chronic ankle instability in controlled research: a position statement of the international ankle consortium. J. Athl. Train. 49, 121–7. 10.4085/1062-6050-49.1.14 24377963 PMC3917288

[B15] He S.-. Q Dum R. P. (1995). Strickia2a3 PL. Topographic organization of corticospinal projections from the frontal lobe: motor areas on the medial surface of the hemisphere. J. Neurosci. 5, 3284–3306. 10.1523/JNEUROSCI.15-05-03284.1995 PMC6578253 7538558

[B16] Hiller C. E. Refshauge K. M. Bundy A. C. Herbert R. D. Kilbreath S. L. (2006). The cumberland ankle instability tool: a report of validity and reliability testing. Arch. Phys. Med. Rehabil. 87, 1235–1241. 10.1016/j.apmr.2006.05.022 16935061

[B17] Huynh V. Lütolf R. Rosner J. Luechinger R. Curt A. Kollias S.. (2022). Descending pain modulatory efficiency in healthy subjects is related to structure and resting connectivity of brain regions. Neuroimage. 247, 118742. 10.1016/j.neuroimage.2021.118742 34863962

[B18] Jiang D. Ao Y. Jiao C. Xie X. Chen L. Guo Q.. (2018). Concurrent arthroscopic osteochondral lesion treatment and lateral ankle ligament repair has no substantial effect on the outcome of chronic lateral ankle instability. Knee Surgery, Sport Traumatol Arthrosc 26, 3129–3134. 10.1007/s00167-017-4774-5 29138920

[B19] Kapreli E. Athanasopoulos S. Papathanasiou M. Van Hecke P. Kelekis D. Peeters R.. (2007). Lower limb sensorimotor network: issues of somatotopy and overlap. Cortex. 43, 219–232. 10.1016/S0010-9452(08)70477-5 17405668

[B20] Klasan A. Putnis S. E. Grasso S. Kandhari V. Oshima T. Parker D. A.. (2021). Tegner level is predictive for successful return to sport 2 years after anterior cruciate ligament reconstruction. Knee Surgery, Sport Traumatol Arthrosc. 29, 3010–3016. 10.1007/s00167-020-06335-4 33118063 PMC8384787

[B21] Krishnan V. S. Shin S. S. Belegu V. Celnik P. Reimers M. Smith K. R.. (2019). Multimodal evaluation of TMS - Induced somatosensory plasticity and behavioral recovery in rats with contusion spinal cord injury. Front. Neurosci. 13, 387. 10.3389/fnins.2019.00387 31068784 PMC6491761

[B22] Lin W. Zhu Q. Gao W. Chen Y. Toh C. H. Styner M.. (2008). Functional connectivity MR imaging reveals cortical functional connectivity in the developing brain. Am. J. Neuroradiol. 29, 1883–1889. 10.3174/ajnr.A1256 18784212 PMC2583167

[B23] Liu H. Cai W. Xu L. Li W. Qin W. (2020). Differential reorganization of SMA subregions after stroke: a subregional level resting-state functional connectivity study. Front Hum. 13, 468. 10.3389/fnhum.2019.00468 32184712 PMC7059000

[B24] Lu H. Kobilo T. Robertson C. Tong S. Celnik P. Pelled G.. (2015). Transcranial magnetic stimulation facilitates neurorehabilitation after pediatric traumatic brain injury. Sci. Rep. 5, 14769. 10.1038/srep14769 26440604 PMC4594036

[B25] Lu M. T. Preston J. B. Strick P. L. (1994). Interconnections between the prefrontal cortex and the premotor areas in the frontal lobe. JCN. 341, 375–392. 10.1002/cne.903410308 7515081

[B26] McCriskin B. J. Cameron K. L. Orr J. D. Waterman B. R. (2015). Management and prevention of acute and chronic lateral ankle instability in athletic patient populations. World J. Orthop. 6, 161–171. 10.5312/wjo.v6.i2.161 25793157 PMC4363799

[B27] Needle A. R. Lepley A. S. Grooms D. R. (2017). Central nervous system adaptation after ligamentous injury: a summary of theories, evidence, and clinical interpretation. Sport Med. 47, 1271–1288. 10.1007/s40279-016-0666-y 28005191

[B28] Ogawa S. Lee T. M. Barrere B. (1993). The sensitivity of magnetic resonance a rat brain to changes in the cerebral oxygenation. Magn. Reson. Med. 29, 205–210. 10.1002/mrm.1910290208 8429784

[B29] Ogawa S. Lee T. M. Kay A. R. Tank D. W. (1990). Brain magnetic resonance imaging with contrast dependent on blood oxygenation (cerebral blood flow/brain metabolism/oxygenation). Proc. Natl. Acad. Sci. U S A. 87, 9868–72. 10.1073/pnas.87.24.9868 2124706 PMC55275

[B30] Osumi M. Sumitani M. Nishi Y. Nobusako S. Dilek B. Morioka S.. (2021). Fear of movement-related pain disturbs cortical preparatory activity after becoming aware of motor intention. Behav. Brain Res. 411, 113379. 10.1016/j.bbr.2021.113379 34051229

[B31] Paulus M. P. Stein M. B. (2010). Interoception in anxiety and depression. Brain Struct. Funct. 214, 451–463. 10.1007/s00429-010-0258-9 20490545 PMC2886901

[B32] Pelletier R. Higgins J. Bourbonnais D. (2015). Is neuroplasticity in the central nervous system the missing link to our understanding of chronic musculoskeletal disorders? BMC Musculoskelet. Disord. 16, 25. 10.1186/s12891-015-0480-y 25887644 PMC4331171

[B33] Ploghaus A. Narain C. Beckmann C. F. Clare S. Bantick S. Wise R.. (2001). Exacerbation of pain by anxiety is associated with activity in a hippocampal network. J. Neurosci. 21, 9896–9903. 10.1523/JNEUROSCI.21-24-09896.2001 11739597 PMC6763058

[B34] Safran M. R. Benedetti R. S. Bartolozzi A. R. Mandelbaum B. R. (1999). Lateral ankle sprains: a comprehensive review part 1: etiology, pathoanatomy, histopathogenesis, and diagnosis. Med. Sci. Sports Exerc. 31, S429–37. 10.1097/00005768-199907001-00004 10416544

[B35] Segerdahl A. R. Mezue M. Okell T. W. Farrar J. T. Tracey I. (2015). The dorsal posterior insula subserves a fundamental role in human pain. Nat. Neurosci. 18, 499–500. 10.1038/nn.3969 25751532 PMC6783299

[B36] Sharma N. Classen J. Cohen L. G. (2013). Neural plasticity and its contribution to functional recovery. Handb. Clin. Neurol. 110, 3–12. 10.1016/B978-0-444-52901-5.00001-0 23312626 PMC4880010

[B37] Shin S. S. Krishnan V. Stokes W. Robertson C. Celnik P. Chen Y.. (2018). Transcranial magnetic stimulation and environmental enrichment enhances cortical excitability and functional outcomes after traumatic brain injury. Brain Stimul. 11, 1306–1313. 10.1016/j.brs.2018.07.050 30082198 PMC6204305

[B38] Song Y. Li H. Sun C. Zhang J. Gui J. Guo Q.. (2019). Clinical guidelines for the surgical management of chronic lateral ankle instability: a consensus reached by systematic review of the available data. Orthop. J. Sport Med. 7, 232596711987385. 10.1177/2325967119873852 31579683 PMC6757505

[B39] Starr C. J. Sawaki L. Wittenberg G. F. Burdette J. H. Oshiro Y. Quevedo A. S.. (2009). Roles of the insular cortex in the modulation of pain: Insights from brain lesions. J. Neurosci. 29, 2684–2694. 10.1523/JNEUROSCI.5173-08.2009 19261863 PMC2748680

[B40] Thompson C. S. Hiller C. E. Schabrun S. M. (2019). Altered spinal-level sensorimotor control related to pain and perceived instability in people with chronic ankle instability. J. Sci. Med. Sport 22, 425–429. 10.1016/j.jsams.2018.10.009 30442546

[B41] Tung K. C. Uh J. Mao D. Xu F. Xiao G. Lu H.. (2013). Alterations in resting functional connectivity due to recent motor task. Neuroimage. 78, 316–324. 10.1016/j.neuroimage.2013.04.006 23583747 PMC3672369

[B42] Wei D. Yang J. Li W. Wang K. Zhang Q. Qiu J.. (2014). Increased resting functional connectivity of the medial prefrontal cortex in creativity by means of cognitive stimulation. Cortex. 51, 92–102. 10.1016/j.cortex.2013.09.004 24188648

[B43] Wessel K. Zeffiro T. Lou J. S. Toro C. Hallett M. (1995). Regional cerebral blood flow during a self-paced sequential finger opposition task in patients with cerebellar degeneration. Brain. 118, 379–393. 10.1093/brain/118.2.379 7735880

[B44] Woo C. W. Krishnan A. Wager T. D. (2014). Cluster-extent based thresholding in fMRI analyses: pitfalls and recommendations. Neuroimage. 91, 412–419. 10.1016/j.neuroimage.2013.12.058 24412399 PMC4214144

[B45] Xue X. Li S. Li H. Li Q. Hua Y. (2021a). Deactivation of the dorsal anterior cingulate cortex indicated low postoperative sports levels in presurgical patients with chronic ankle instability. BMC Sports Sci. Med. Rehabil. 13, 121. 10.1186/s13102-021-00353-6 34627368 PMC8501719

[B46] Xue X. Zhang Y. Li S. Xu H. Chen S. Hua Y.. (2021b). Lateral ankle instability-induced neuroplasticity in brain grey matter: a voxel-based morphometry MRI study. J. Sci. Med. Sport. 24, 1240–1244. 10.1016/j.jsams.2021.06.013 34281769

[B47] Zhuo M. (2016). Neural mechanisms underlying anxiety-chronic pain interactions. Trends Neurosci. 39, 136–145. 10.1016/j.tins.2016.01.006 26878750

