# Supplementary figures and images for: Neuroplasticity of pain processing and motor control in CAI patients: A UK Biobank study with clinical validation
Source: Front Mol Neurosci. 2023 Feb 14;16:1096930. doi: 10.3389/fnmol.2023.1096930 (PMC9971622; doi:10.3389/fnmol.2023.1096930)

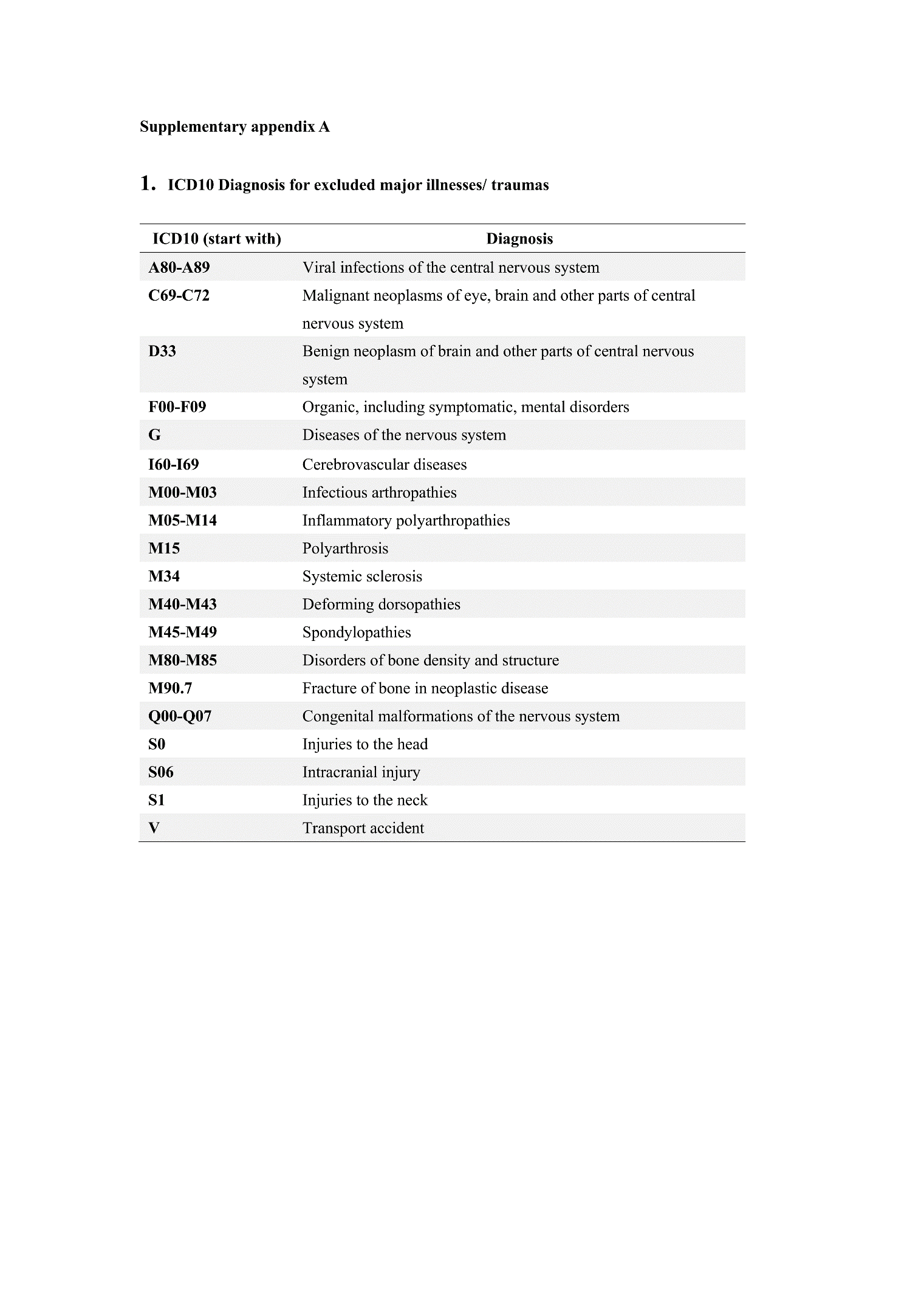

Supplement: Supplementary file 1 [file Image_1.PNG]

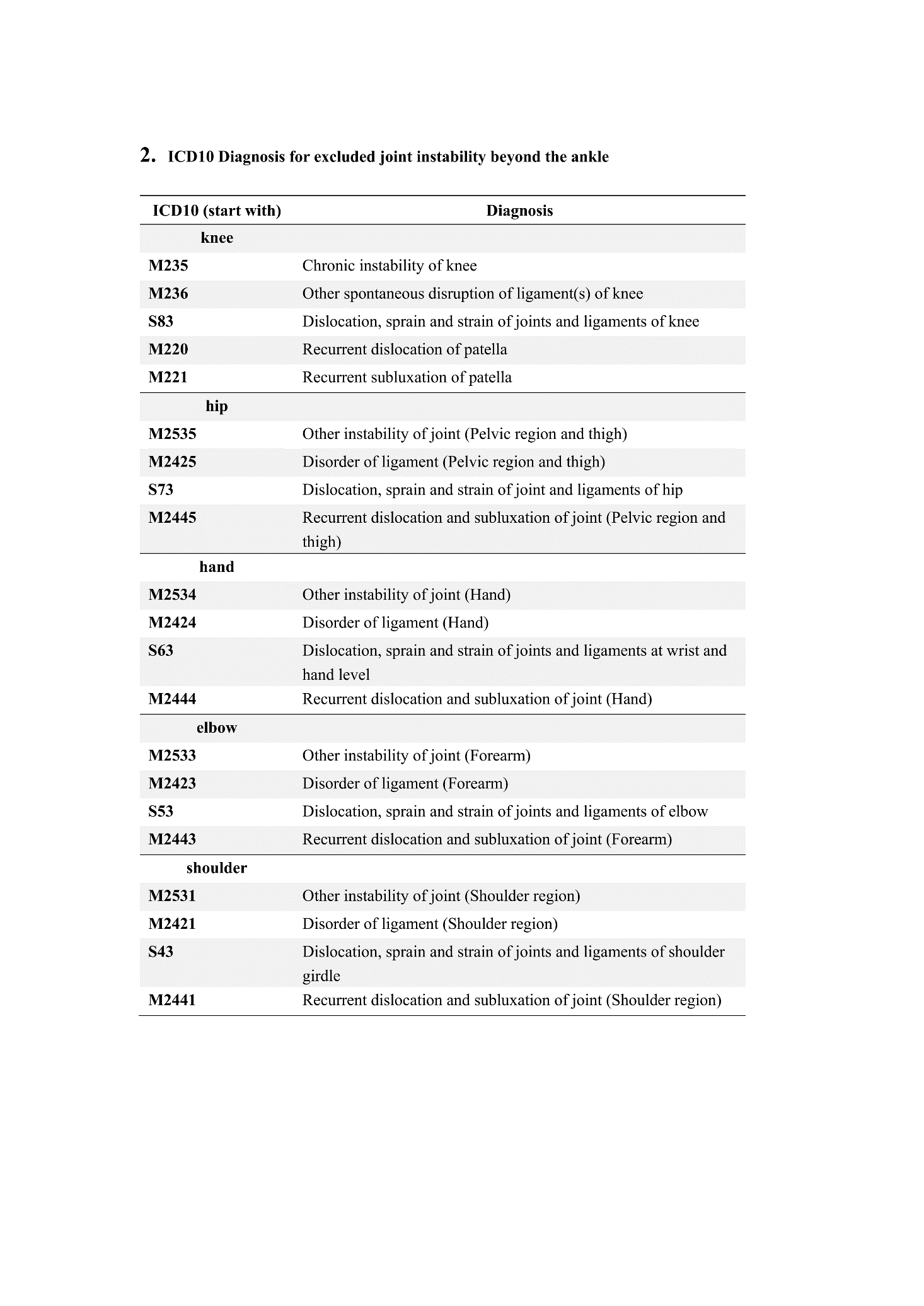

Supplement: Supplementary file 2 [file Image_2.PNG]

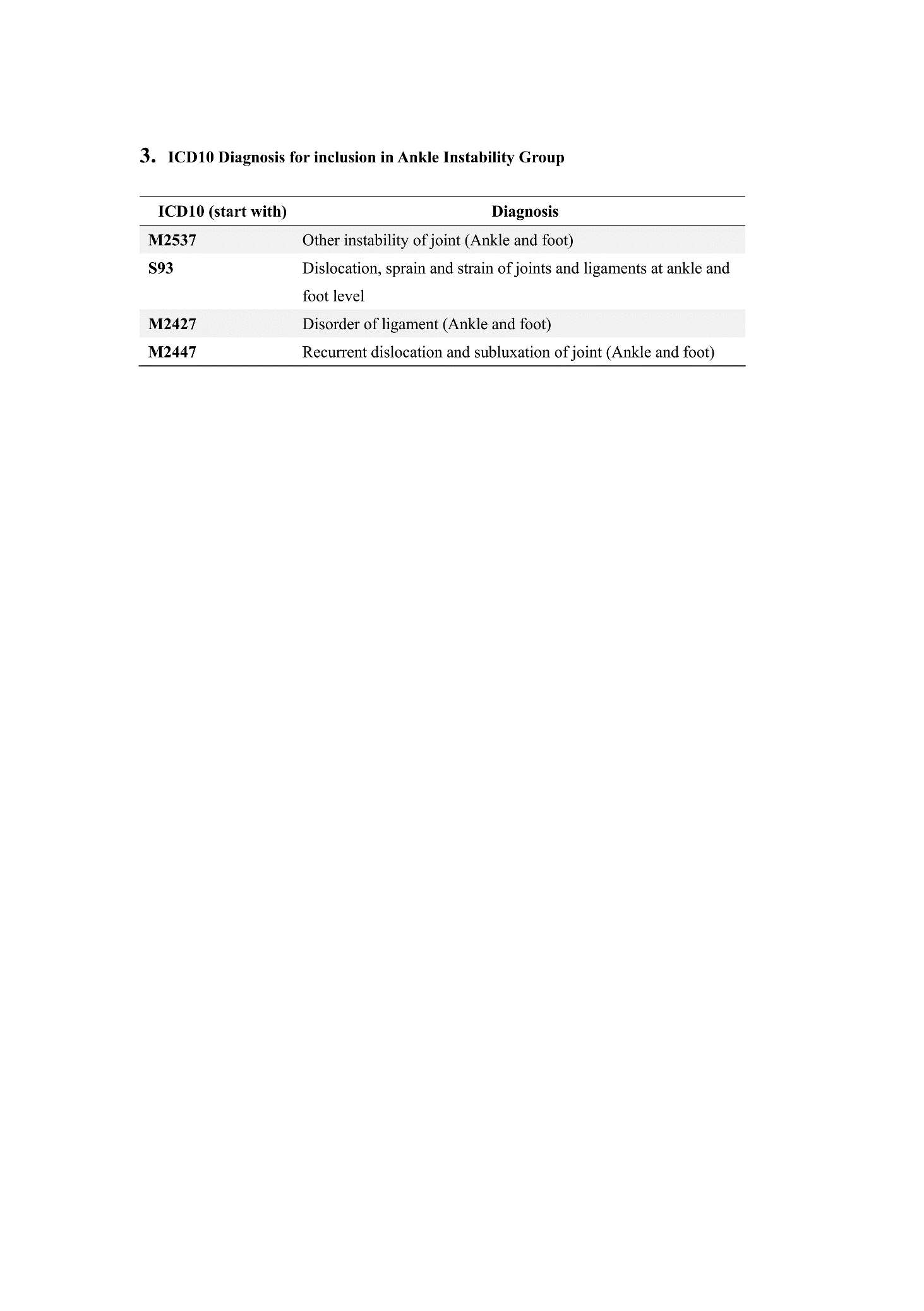

Supplement: Supplementary file 3 [file Image_3.PNG]
